# Perceived Family Stress Predicts Poor Metabolic Control in Pediatric Patients with Type 1 Diabetes: A Novel Triadic Approach

**DOI:** 10.1155/2022/3809775

**Published:** 2022-05-12

**Authors:** Fiona L. Mahler, Daniel Konrad, Markus A. Landolt

**Affiliations:** ^1^Department of Psychosomatics and Psychiatry, University Children's Hospital Zurich, University of Zurich, Switzerland; ^2^Department of Endocrinology and Diabetes, University Children's Hospital Zurich, University of Zurich, Switzerland; ^3^Children's Research Center, University Children's Hospital Zurich, University of Zurich, Switzerland; ^4^Division of Child and Adolescent Health Psychology, Department of Psychology, University of Zurich, Zurich, Switzerland

## Abstract

**Objective:**

Poor metabolic control and low treatment adherence remain major issues for many pediatric patients with type 1 diabetes. Important risk factors for both include psychosocial variables such as stress. To date, stress in type 1 diabetes patients and their parents has been investigated at an individual level. The present study tested the hypothesis that patients', mothers', and fathers' perceived stress is positively related to each other and therefore is a factor common to the family. This factor was then hypothesized to be related to patients' poorer treatment adherence behavior and metabolic control. *Research Design and Methods*. This cross-sectional study at the University Children's Hospital Zurich included 190 type 1 diabetes patients (age: 7–18 years; illness duration: ≥1 year) and their families. The Perceived Stress Scale was used to measure the self-reported stress of patients, mothers, and fathers. Patients' treatment adherence was rated by their endocrinologists. HbA1c served as indicator of metabolic control. A structural equation model (SEM) was conducted for analysis.

**Results:**

The SEM showed adequate model fit. Patients' (*β* = .567, *p* ≤ .001), mother's (*β* = .621, *p* ≤ .001), and father's (*β* = .585, *p* ≤ .001) perceived stress loaded all on a single factor, perceived family stress. This factor was significantly associated with treatment adherence (*β* = −.384, *p* ≤ .001) and with HbA1c (*β* = .210, *p* = .012) of patients.

**Conclusions:**

Results confirmed perceived family stress to be a common family construct. Because perceived family stress might have a negative impact on patients' treatment adherence and HbA1c, subjective stress appraisals of patients and both parents should be considered when counseling children and adolescents with type 1 diabetes.

## 1. Introduction

Management of type 1 diabetes in children and adolescents requires a major effort from both patients and their families. Adherence to the medical regimen is necessary to achieve good metabolic control, which is significantly influenced by psychosocial factors [1]. One of these influencing factors is stress, a factor extensively explored for its consequences on physical and mental health. Because stress is a biopsychosocial construct, its complexity is difficult to be determined. Stress can be measured objectively from a biological perspective (e.g., by stress hormones in hair) or subjectively from a psychological perspective (e.g., by questionnaire) [2, 3].

To better understand why stress is important to type 1 diabetes and its management in children and adolescents, it is important to consider stress as a biopsychosocial construct. From a biological perspective, stress can be linked to the release of stress hormones, which antagonize insulin action [4]. Thus, stress may pose challenges to patients in keeping their blood glucose levels well balanced. In fact, a positive association between self-reported stress and levels of glycated hemoglobin (HbA1c) has been found in children and adolescents with type 1 diabetes [5, 6].

From a psychological perspective, stress is a factor that impacts behavior. For example, type 1 diabetes-related subjective distress in adolescents was shown to be associated with reduced self-care [7] and poor treatment adherence [5]. However, stress has not always been conceptualized in the same way. Whereas previous studies in this field have focused on subjective stress in relation to diabetes and diabetes management, other possible sources of stress have received less attention. There are indications that type 1 diabetes patients do not only experience stress arising directly from the disease and its management. For example, it was shown that adolescents with type 1 diabetes do not experience diabetic stressors as more stressful than everyday stressors [8]. Farrell and colleagues [5] found both diabetes-specific and general stress to be positively related with HbA1c in youths with type 1 diabetes; additionally, general stress was associated with poorer treatment adherence. Another study by Berlin and colleagues [9] identified three different stress profiles in adolescents with type 1 diabetes: “low stress,” “interpersonal/peer,” and “family stress.” The authors investigated if the participants of these three stress groups differ regarding metabolic control. It was found that only the “family stress” group's HbA1c was significantly higher compared to the other groups.

Taken together, children and adolescents experience different sources of stress, which can be related to treatment adherence and metabolic control and therefore should be considered. According to Leventhal's Common Sense Model [10], individual perceptions and coping behavior affect the amount of psychological distress a stressor can cause. Consequently, avoiding a focus on specific stressors and instead focusing on individuals perceived stress, thus how stressed an individual feels, would enable a more comprehensive approach to measuring subjective stress. So far, the relationship between perceived stress and HbA1c or treatment adherence has not received enough attention. To our knowledge, perceived stress was only investigated once in a population of children and adolescents with type 1 diabetes. Rechenberg and colleagues [11] found perceived stress of adolescents with type 1 diabetes to be related with poorer treatment adherence and metabolic control. However, perceived stress has also been shown to be related to unhealthy behavior in general [12].

Because parents are usually involved in the management of type 1 diabetes in their children, their stress experience may be relevant to metabolic control in their children as well. Indeed, higher subjective parenting stress has been shown to be associated with poorer metabolic control [13]. However, whereas most studies have focused on parenting stress, only one study has yet investigated the association between perceived stress in caregivers and poorer metabolic control, and it found the association to be negative [14].

To summarize, research to date has focused on subjective diabetes-related stress and parenting stress rather than perceived stress. In addition, no research has been found that included both the subjective stress of type 1 diabetes patients and of their parents. Moreover, the research that focused on parents' subjective stress mainly investigated mothers (85–97%), with little attention paid to fathers.

In the present study, we shifted the focus from specific stressors to the individuals' responses to these stressors by investigating the subjective construct of perceived stress [15]. We aimed first to investigate whether patients', mothers', and fathers' perceived stress is positively related to each other within a family and therefore is not an individual factor but a factor common to the family. Second, we hypothesized that higher perceived stress within a family is related to poorer treatment adherence behavior and metabolic control in the patient. Finally, we wanted to check all these assumptions together in a single structural equation model.

## 2. Research Design and Methods

### 2.1. Participants and Procedure

Participants were recruited consecutively at the University Children's Hospital Zurich during routine outpatient visits. All German-speaking pediatric patients aged between 7 and 18 years diagnosed with type 1 diabetes at least one year before recruitment and their caregivers were invited to participate. Patients with developmental disorders with impact on the diabetes management were excluded. Of 223 eligible patients and caregivers, in 197 (88%) cases, at least one family member consented and participated. In cases of participating siblings, one of them was selected randomly by coin toss; seven patients were excluded in this way. To check if there was a selection bias, we calculated an independent *t*-test. The selected siblings did not differ from the excluded siblings regarding HbA1c, treatment adherence, and perceived stress. Overall, data from 190 families were used in the analyses. Of the 190 participating patients, 85 (44.7%) were female, and 105 (55.3%) were male. Mean age was 14.2 (SD = 3.1) years.

In this cross-sectional study, the children and adolescents took part in structured interviews (*N* = 179), whereas their parents (*N*_mothers_ = 143, *N*_fathers_ = 121) completed questionnaires. The interviews were conducted by trained interviewers with a background in psychology at the University Children's Hospital Zurich in the immediate aftermath of a routine outpatient visit. In four cases, interviews were conducted 0–15 days after the routine outpatient visit at the patients' homes. Questionnaires were given to the parents at the same time as the interview was conducted. In addition, medical data from all the 190 participants were extracted from their medical records and collected from attending pediatric endocrinologists. The current study was approved by the Ethics Commission of Canton Zurich (BASEC-Nr. 2018-00374).

### 2.2. Measures

#### 2.2.1. Glycemic Control

Glycemic control was assessed by HbA1c measurement. Participants' HbA1c levels were measured during routine outpatient visits at the University Children's Hospital Zurich using a DCA Vantage Analyzer (Siemens, Munich, Germany). The research team had access to the medical records in which individuals' HbA1c measurements were available. HbA1c levels, measured the same day as the interview was taken, were used for statistical analyses.

#### 2.2.2. Perceived Stress Scale

The German version of the Perceived Stress Scale (PSS-10) [3, 16] was used to assess the self-reported stress of patients, their mothers, and their fathers. This 10-item questionnaire measures the extent to which individuals perceive their lives to be uncontrollable and how overloaded they feel. Items were rated on 5-point Likert scales. The PSS-10 is a widely used instrument with good internal consistency and construct validity [16]. In the current study, internal consistency was good for all three samples (patients: *α* = .80; mothers: *α* = .85; fathers: *α* = .84).

#### 2.2.3. Treatment Adherence

To measure patients' treatment adherence in the context of type 1 diabetes management, a 6-item questionnaire was constructed by the authors. More specifically, five aspects of treatment adherence were assessed: (1) following diet, (2) measuring blood sugar, (3) taking insulin shots, (4) keeping glucose diary updated, and (5) appearing regularly to appointments. In addition, the sixth question assessed the overall treatment adherence of patients. The questions were rated on a 3-point Likert scale by the patients' pediatric endocrinologist: 0 = ^“^yes,^”^1 = ^“^sometimes,^”^ and 2 = ^“^no.^”^ The sixth question could be answered by 0 = ^“^good,^”^1 = ^“^medium,^”^ and 2 = ^“^poor.^”^ Because this instrument was newly constructed, the structure of the treatment adherence questionnaire was determined with an exploratory factor analysis. Both the Bartlett test (*χ*^2^(15) = 634.93, *p* < .001) and the Kaiser-Meyer-Olkin Measure of Sampling Adequacy (KMO = .83) indicated eligibility for factor analysis. A principal component analysis was calculated with varimax rotation. One factor with eigenvalue >1 was identified. Thus, the factor analyses indicated all six items loading on the same factor. Internal consistency was acceptable (*α* = .77).

### 2.3. Statistical Analyses

First, participant characteristics were computed, and we checked for sex differences in treatment adherence, HbA1c, and perceived stress with univariate analyses of variance (ANOVA). The hypotheses were tested in two steps. First, bivariate relationships between study variables were investigated by conducting Pearson correlations, except for relationships with dichotomous variables which were calculated by point-biserial correlations. All these analyses were performed with IBM SPSS statistics version 27. Second, a structural equation model (SEM) was conducted with Mplus version 8.6. We used the full information maximum likelihood procedure due to its advantages over classical methods [17]. To assess model fit, standard model fit indices were used: normed *χ*^2^ (*χ*^2^ value/degree of freedom) < 5, root mean square of approximation (RMSEA) < .08, standardized root mean square residual (SRMR) < .1, and comparative fit index (CFI) > .9 [18, 19]. To control for age and illness duration, we added these variables to the SEM.

## 3. Results

### 3.1. Sample Characteristics

Information about sociodemographics and illness-related variables are presented in Table 1.

### 3.2. Preliminary Analyses

To check for sex differences in treatment adherence, HbA1c, and perceived stress, univariate ANOVAs were calculated (see Table 2). There were no significant sex differences in treatment adherence or metabolic control. However, female patients reported higher perceived stress than male patients. The size of this effect can be considered small [20].

Bivariate relationships (see Table 3) showed negative bivariate associations for treatment adherence with HbA1c levels, patients' age, and perceived stress of patients and mothers. HbA1c levels were positively related to the perceived stress of patients and mothers. Further, perceived stress levels within families were all positively related. The difference between children and adolescents living with both their parents and those not living with both their parents did not correlate with patients' treatment adherence, HbA1c, and perceived stress and fathers' perceived stress. However, a positive relationship with mothers' perceived stress was found.

### 3.3. Structured Equation Model

**Figure 1:**
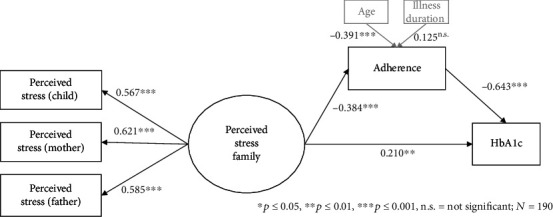
Structural equation model (SEM) with latent factor perceived family stress and the associations between perceived family stress and patients' treatment adherence, perceived family stress, and patients' glycated hemoglobin (HbA1c), patients' treatment adherence, and patients' HbA1c were calculated. To control for patients' age and illness duration, their association with patients' treatment adherence were also computed. All coefficients are standardized.

The results of the SEM are illustrated in Figure 1. The SEM showed adequate model fit (normed *χ*2: 32.473/12 = 2.706, *p* ≤ .001; RMSEA: .095, *p* = .030; SRMR: .067; CFI: .914). The indicator variables for perceived stress of patients (*β* = .567, *p* ≤ .001), of mothers (*β* = .621, *p* ≤ .001), and of fathers (*β* = .585, *p* ≤ .001) loaded significantly on the latent factor. Thus, the latent factor of perceived family stress was confirmed. All of the regression analyses within the SEM were statistically significant. The latent factor of stress within the family was positively associated with HbA1c values (*β* = .210, *p* = .012) and negatively with treatment adherence (*β* = −.384, *p* ≤ .001). Treatment adherence was negatively associated to HbA1c values. Whereas age was negatively associated to treatment adherence, illness duration was positively associated with treatment adherence.

## 4. Discussion

The present study is the first to focus on perceived stress as a risk factor of poor treatment adherence and metabolic control not solely in children and adolescents with type 1 diabetes or separately in their parents but with a triadic approach in patients, mothers, and fathers together. The findings clearly support the need to focus on the perceived stress of the whole family, rather than the perceived stress of individuals, to fully understand the associations of stress with treatment adherence and metabolic control.

Female patients reported higher perceived stress than male patients; this contrasts with most previous studies, which did not find such sex differences [7]. No sex differences were found for treatment adherence and HbA1c, which is in line with many studies (e.g., [5, 21, 22]). Furthermore, our analyses showed negative bivariate relationships for treatment adherence with HbA1c levels, patients' age, and perceived stress of patients and mothers. HbA1c levels were positively related with perceived stress of patients and mothers. Interestingly, father's perceived stress was not related to treatment adherence or HbA1c. Because most research in this field focused on mothers or caregivers in general and investigated parenting stress rather than perceived stress, it is difficult to compare this result. A possible explanation would be that in the present sample, fathers stated to do more work other than homemaking compared to mothers. Therefore, fathers might have spent less time with their children and might be less involved in the type 1 diabetes management. However, perceived stress levels within families were all positively related. This strengthens our hypothesis that perceived stress may be interpreted as a factor at the family level rather than at the individual level. Further, there was no difference in patients' treatment adherence, HbA1c, and perceived stress between children and adolescents living with both their parents and those who did not live with both their parents. However, mothers' but not fathers' perceived stress was significantly higher for patients not living with both their parents. Because of that finding, it was checked how many mothers and fathers are not in a romantic relationship at all. About half of mothers not living with the father of the child stated to be in a relationship, most of them living together. Whereas about two-thirds of fathers not living with the child's mother stated to be in a new relationship, most of them living together. So, mothers not living with patients' fathers were more often single than fathers not living with patients' mothers. It is comprehensible that being a single parent can be very challenging, which could explain here why mothers' but not fathers' perceived stress is related to the variable living situation.

The results of the main analysis, the SEM, support our hypotheses. The model fit indices of the SEM showed good fit except for the RMSEA (.095), for which we suggested < .08 as a good model fit. But because a poor model fit for RMSEA would be considered ≥ .1, our findings can still be categorized as adequate [18].

The SEM confirmed the latent triadic factor perceived family stress. This means that perceived stress is to be considered a factor common to the family rather than an individual factor. This may be because perceived stress is dependent on stressful life events. Given the fact that the most stressful life events, such as the death of a loved one or divorce, usually concern the whole family, it is comprehensible that family members' perceived stress is associated with each other's [23]. However, individuals' perceptions of the stressfulness of an event are affected by their cognitive appraisals of it [24]. These appraisals are influenced by individuals' characteristics, for example, optimism or belief in control, which again might be affected by their parents' characteristics [24]. Furthermore, Doron et al. [12] found that individuals with maladaptive coping styles experience higher levels of perceived stress than those with adaptive coping styles. Since parents' coping styles can have an impact on their children's, parents', and children's perceived stress levels may well also be related to each other [25]. Another explanation is that children might have similar physiological reactions to stress and therefore a similar stress experience to their parents due to the biological connection. Saxbe et al. [26] found patterns of physiological influence of stress within families. More precisely, they found positive associations in saliva cortisol levels, a biomarker for stress, between mothers, fathers, and their children or adolescents over multiple timepoints. Of course, the previous two arguments can only explain the relationships between children and their biological parents, but not the relationships between parents. In the present study, the questionnaires information on who answered the PSS-10 did not differentiate between a biological and a step-parent. In cases of patients not living with both their biological parents, it was thus not possible to differentiate whether a step-parent or a biological parent not living with the child answered the questionnaire.

Other concepts that are important to consider are the spillover and crossover effects of stress within a family. The spillover effect means that a stressed person exhibits more negative behavior, sees more problems in a relationship, and uses a more negative attributional style, such as blaming the partner [27]. The crossover effect means that the stress of an individual may have an interpersonal effect on the emotions of other family members [27]. For example, husbands' work stress has been shown to be related to higher levels of psychological distress in wives [28]. To sum up, there are several possible explanations for our finding that perceived stress is a factor common to the family rather than an individual factor. However, in this research field, stress has never been taken into account as a common family factor.

In support of our hypothesis, the results show that the latent factor perceived family stress was positively associated with HbA1c levels and negatively associated with treatment adherence. This means that patients with higher perceived family stress show less treatment adherence, according to their pediatric endocrinologists. Moreover, they have significantly higher HbA1c levels. These results are in line with existing literature that found that higher stress levels in pediatric patients with type 1 diabetes are associated with lower treatment adherence and higher HbA1c. However, past research focused mainly on diabetes-related distress instead of perceived stress. A study by Berlin et al. [9] identified three stress profiles in adolescents with type 1 diabetes: “low stress,” “interpersonal/peer stress,” and “family stress.” Berlin et al. [9] investigated whether the participants of these three stress groups differ in metabolic control. They found that only the family stress group's HbA1c was significantly higher than the other groups' HbA1c. Consequently, factors other than illness-related subjective stress were also found to be relevant to metabolic control. Furthermore, Farrell et al. [5] showed that general subjective stress is also negatively related to adherence behavior and metabolic control in adolescents. However, the significance of the perceived stress levels of the whole family has never been analyzed. Our results indicate the relevance of mothers' and fathers' perceived stress as well as patients' adherence to type 1 diabetes management and metabolic control.

### 4.1. Strengths and Limitations

A strength of this study is its highly representative sample, thanks to the high participation rate of over 85%. Notably, nonparticipants did not differ from participants in HbA1c or age. In addition, a high proportion of fathers participated in this study, which allowed us to analyze data with a novel triadic approach. To our knowledge, no previous study had collected enough data from fathers to examine this issue. Further, parents' characteristics in our sample are quite representative for the population in Switzerland, for example, in education [29].

A limitation of the study is the lack of data for older patients. For example, adolescents were not always accompanied by their parents on visits to the doctor, which made it harder for us to approach them. Moreover, we have fewer treatment adherence ratings from pediatric endocrinologists for younger children, because in some of these cases the main responsibility for the diabetes management was borne by the parents. Another limitation is that we do not know whether the parent questionnaires were answered by the biological parent or the step-parent because this was not separately assessed. Another limitation is the cross-sectional design of the study, which does not allow causal conclusions. Therefore, the direction of the effects found was only inferred from theory.

### 4.2. Implications for Research and Clinical Management

Our results suggest that pediatric endocrinologists should consider both patients' and parents' perceived stress when counseling children and adolescents with type 1 diabetes. It is important to ask parents about their perceived stress, and parents need to understand that the stress they perceive is not independent of the stress perceived by other family members. It is important for parents to know that the stress they perceive may have a negative impact on their children's metabolic control. With this knowledge, parents might be more willing to participate in stress management training or family counseling. In fact, some studies have investigated stress-reducing interventions for type 1 diabetes patients or their parents. For example, Tsiouli et al. [30] found that parents' perceived stress could be significantly decreased by an 8-week relaxation intervention. However, they did not check for effects on patients' metabolic control or treatment adherence. Therefore, it would be helpful if further research investigating stress reduction interventions for parents of children and adolescents with type 1 diabetes also examined their effects on patients' metabolic control. A study by Attari et al. [31] conducted 3-month stress management training in adolescents and young adults with type 1 diabetes and found significantly better metabolic control in participants after the training than before. However, several studies have investigated the effect of a stress reduction intervention in type 1 diabetes patients without finding any effect on metabolic control. To summarize, more studies are needed to determine what kind of stress reduction intervention is more effective in reducing patients' and parents' perceived stress and therefore in improving patients' metabolic control.

## Figures and Tables

**Table 1:** Participants' characteristics.

HbA1c^a^		
%		7.9% (1.5)
(mmol/mol)		63.3 (16.8)
Sex^b^		
Girls		85 (44.7%)
Boys		105 (55.3%)
Other		0 (0%)
Age (years)^a^		14.2 (3.1)
Age at diagnosis (years)^a^		7.7 (4.0)
Illness duration (years)^a^		6.5 (3.9)
Insulin administration^b^		
Injection		165 (86.8%)
Pump		23 (12.1%)
Other/missing		2 (1.0%)
Living situation		
Lives with both biological parents		109 (57.4%)
Does not live with both biological parents		42 (22.1%)
Missing		39 (20.1%)
Participation		
Both parents and child		107 (56.3%)
Both parents no child		4 (2.1%)
Mother and child		27 (14.2%)
Father and child		8 (4.2%)
Only mother		5 (2.6%)
Only father		2 (1.1%)
Only child		37 (19.5%)
	Mothers (*N* = 190)	Fathers (*N* = 190)
Education ^b^		
University	36 (18.9%)	44 (23.2%)
High school	33 (17.4%)	35 (18.4%)
Vocational education (3-4 years)	55 (28.9%)	46 (24.2%)
Vocational education (1-2 years)	7 (3.7%)	6 (3.2%)
Primary education	12 (6.3%)	14 (7.4%)
Unqualified	4 (2.1%)	2 (1.1%)
Missing	43 (22.6%)	43 (22.6%)
	Mothers (*N* = 190)	Fathers (*N* = 190)
Occupation		
100% homemaker ^b^	35 (18.4%)	4 (2.1%)
Work (other than homemaker)^b^	109 (57.4%)	136 (71.6%)
-Work time (%)^a^	61.5 (22.8)	96.6 (10.4)
Unemployed, looking for work ^b^	3 (1.6%)	3 (1.6%)
Missing	43 (22.6%)	47 (24.7%)

Note: *N* = 190. ^a^Mean (SD). ^b^N (%).

**Table 2:** Univariate ANOVAs for sex.

Variable	Female^a^	Male^a^	*F* value	*p* value	*η* ^2^
HbA1c					
%	7.9 (1.5)	8.0 (1.6)	.105 (df = 1; 188)	.746	.001
(mmol/Mol)	62.9 (16.3)	63.7 (17.3)			
Treatment adherence	10.2 (2.7)	9.6 (3.7)	1.310 (df = 1; 174)	.254	.007
PS child	15.9 (5.9)	13.8 (6.0)	5.430 (df:1; 177)	.021	.030

^a^Mean (SD); PS = perceived stress.

**Table 3:** Bivariate relationships between patients' age, illness duration, metabolic control, treatment adherence, and perceived stress, and mothers' and fathers' perceived stress, and living situation.

		1.	2.	3.	4.	5.	6.	7.	8.
1.	Age		.353∗∗∗	.136	-.339∗∗∗	.109	-.065	.063	-.038
2.	Illness duration			.068	.005	-.035	-.034	.106	-.106
3.	HbA1c				-.737∗∗∗	.360∗∗∗	.227∗∗	.151	.048
4.	Treatment adherence					-.348∗∗∗	-.242∗∗	-.033	.020
5.	PS child						.305∗∗∗	.303∗∗∗	-.037
6.	PS mother							.456∗∗∗	.196∗
7.	PS father								-.057
8.	Living situation^a^								

∗*p* ≤ .05, ∗∗*p* ≤ .01, and ∗∗∗*p* ≤ .001. ^a^Dichotomous variable: child lives with both their parents (0) or not (1). PS = perceived stress.

## Data Availability

The data that support the findings of this study are available from the corresponding author upon reasonable request.
